# Comparative effectiveness analysis of Medicare dialysis facility survey processes

**DOI:** 10.1371/journal.pone.0216038

**Published:** 2019-04-26

**Authors:** Sehee Kim, Fan Wu, Claudia Dahlerus, Deanna Chyn, Yi Li, Joseph M. Messana

**Affiliations:** 1 Department of Biostatistics, University of Michigan, Ann Arbor, Michigan, United States of America; 2 Biogen Inc., Cambridge, Massachusetts, United States of America; 3 Kidney Epidemiology and Cost Center, University of Michigan, Ann Arbor, Michigan, United States of America; 4 Department of Public Health Sciences, University of Virginia, Charlottesville, Virginia, United States of America; 5 Division of Nephrology, Department of Internal Medicine, University of Michigan, Ann Arbor, Michigan, United States of America; National Institute of Health and Nutrition, National Institutes of Biomedical Innovation, Health and Nutrition, JAPAN

## Abstract

**Background:**

To assure and improve the quality and safety of care provided by dialysis facilities, federal oversight has been conducted through periodic survey assessment. However, with the growing number of individuals living with ESRD and dialysis facilities, state survey agencies have faced challenges in time and resources to complete survey activities. Therefore, the survey process (‘Basic Survey’ used prior to 2013) was redesigned in order to develop a more efficient process (‘Core Survey’ newly implemented since 2013). The purpose of this analysis was to evaluate and compare dialysis facility survey outcomes between the Core and Basic Survey processes, using a causal inference technique. The survey outcomes included condition-level citations, total citations (condition- and standard-level), and citation rate per survey-hour.

**Methods:**

For comparisons of non-randomly assigned survey types, propensity score matching was used. Data were drawn from CMS’ Quality Improvement Evaluation System (QIES) database from January 1, 2013 through July 31, 2014. Covariates available included survey type, facility characteristics (state, urban, practices catheter reuse, dialysis modalities offered, number of patients, mortality, hospitalization, infection) and survey-related characteristics (number of surveyors, time since last survey).

**Results:**

Compared to the Basic Survey, the Core Survey identified 10% more total citations (P = 0.001) and identified condition-level citations more frequently, although the latter finding did not reach statistical significance. These findings suggest an increase of 10% in citation rate (i.e. ratio between citations and survey time) with the Core survey process (P = 0.002).

**Conclusions:**

Greater efficiency has implications for attenuating the time-intensive burden of the state survey process, and improving the safety and quality of care provided by dialysis facilities.

## Introduction

### Medicare dialysis oversight (1991–2011)

The Medicare cost of treating end-stage renal disease (ESRD) in the U.S. has increased dramatically over the past 20 years, from less than $10 billion in 1991 to $32.9 billion for care in 2012. This increase is the result of both the rising costs of care as well as the growing number of individuals living with ESRD, with the number of people with ESRD rising from 186,261 in 1991 to 678,383 in 2014 [1–2]. As a result, the quality and safety of care in this situation has been a concern for policy makers, consumers and some providers. Similar to other healthcare settings (e.g., nursing homes), regulation of the quality and safety of care provided by dialysis facilities participating in the Medicare ESRD program has been conducted through State Survey Agencies under contract with the Center for Medicare and Medicare Services (CMS). The periodic survey assesses safety and quality of dialysis care to determine whether facilities are compliant with federally mandated standards, the ESRD *Conditions for Coverage* (CfC) regulations (Conditions for Coverage for End-Stage Renal Disease Facilities) [3]. In 2008, a substantially revised ESRD CfC was implemented, which resulted in numerical expansion of and increased detail for the CfC requirements. The 2008 Medicare dialysis facility CfC increases the emphasis on quality assessment and performance improvement, focusing on process improvement, which reflects more recent advances in the quality improvement field [4]. Specific issues changed by the revised CfC include expanded standards for water quality and new rules on technician, nurse manager, medical director, and dietician credentials, as well as more emphasis on interdisciplinary care and self-management/patient involvement in his/her own care.

All dialysis facilities applying for participation in Medicare’s ESRD Program undergo an initial survey to receive Medicare certification when they begin providing dialysis care. State agencies conduct recertification and complaint-prompted surveys thereafter. Those facilities failing to meet the conditions and standards included in the CfC receive deficiency citations. Violations of the regulations are classified either as condition-level citations (serious citations, that if uncorrected, warrant withdrawal of Medicare certification or closure) or standard-level citations (less serious, without threat of closure).

### Limited resources available for ESRD surveys

The increased ESRD patient population changed the landscape of dialysis care during the 1990s and in the early 2000s, which saw a rise in for-profit facilities and an expansion of corporate chain providers [2,5]. State survey agencies faced challenges keeping pace with the increased number of facilities. In response, the federal government nearly tripled its funding for dialysis facility survey activities, from $3.1 million in fiscal year (FY) 1998 to $8.2 million in FY 2002 [5–6]. In this context, implementation of the revised CfC regulations could enhance efforts at continuing to provide more comprehensive oversight of dialysis facilities for quality improvement. However, at the same time, the added burden of evaluating dialysis facilities for compliance with the expanded CfC could potentially stretch already limited state survey agency resources and the ability to meet federal requirements for survey frequency. The survey process is resource intensive, typically requiring two to three days, or more, of surveyor and dialysis facility staff time per survey. The added burden of the expanded CfC in the context of national dialysis program growth created an urgent need for development of a more efficient dialysis facility survey process for use in this federal program.

### Core Survey process

In FY 2012, the Survey and Certification Group (SCG) in CMS’ Center for Clinical Standards and Quality embarked on a redesign of the dialysis facility survey process, developing the Core Survey as a focused process of the existing full Basic Survey. A team of subject matter experts (experienced dialysis facility surveyors, dialysis healthcare providers and ESRD Network personnel) reviewed the existing survey process and identified survey areas and specific survey questions considered to be most critical to ensuring dialysis patient safety and quality of care. These questions represent approximately 1/3 of the ESRD survey citations (i.e., “tags” or “findings”) represented in the Basic Survey. Within each survey task, they identified a core set of “trigger” tags associated with this refined question set that, if identified, would require the survey team to expand the Core Survey into a Basic Survey. The Core Survey differs from the Basic Survey in three ways: focus on infection prevention and control by utilizing the existing infection control Checklists; thoroughly assess each facility’s Quality Assurance and Performance Improvement (QAPI) program; and use facility-specific, data-driven information to focus surveyor activities in selected areas. The on-site activities of the Core Survey represent a more focused set of activities compared to the Basic Survey. For example, under the Core Survey, surveyors use a selected group of facility clinical outcomes, such as vascular access use, anemia management, fluid management and care coordination with hospitals and other providers, to identify areas in which the dialysis facility is performing at or above the national average. This level of performance is considered evidence that the underlying structures and processes needed to achieve clinical outcomes in these focused areas are adequate to meet the CfC requirements. Other areas of the CfC (e.g. water/dialysate quality, infection control, QAPI, medical record review) are also evaluated in all Core Surveys. Please see S1 Table for more details on the changes between the Basic and Core process.

Collectively, the focused and prescriptive approach of the Core Survey is intended to increase the effectiveness and efficiency of inspections to identify facilities out of compliance with the CfCs. This is particularly important given the growing numbers of people with ESRD and dialysis facilities. In this study, the goal is to compare the efficiency of the two survey processes: the Core Survey (implemented in 2013) and the Basic Survey (in use prior to 2013) in terms of the number of citations identified per time spent on-site.

## Materials and methods

### Study design and setting

A quasi-experimental study design was used to examine the effects of the Core survey on the outcomes, relative to the Basic survey. Such a study design is commonly used to estimate the causal impact of an experiment in an observational setting, but lacks the element of random assignment to experimental arms as opposed to the traditional randomized control trials. We will address the lack of randomization (i.e., reduce the bias due to confounding) in our statistical analysis by using the propensity score approach [7], a commonly used causal inference technique.

CMS developed the Core survey process over the first six months of 2012; this development period was followed by a three-month pilot testing phase in 11 volunteer states. In early FY 2013, SCG began training state surveyors on the use of the Core survey, regardless of whether they were in a volunteer state. CMS required all surveyors who had undergone the training to use the Core survey process. Therefore for our analysis, we used the first dialysis facility survey conducted during the study period from January 1, 2013 through July 31, 2014.

### Data sources

We obtained dialysis facility survey data from CMS’ national administrative survey database, the Quality Improvement Evaluation System (QIES). This database houses information on various aspects of surveys conducted in all healthcare settings. Data elements included the survey type, the number of hours each survey team member spent on the survey and which citations (condition- or standard-level) issued to the facility. We identified facility characteristics and patient outcomes from an extensive national ESRD patient database, which is primarily based on the CMS Consolidated Renal Operations in a Web-enabled Network (CROWN) system. Patient de-identified data were used in the analyses for the manuscript. Access to the data was allowed under the general quality improvement/oversight waiver associated with federal government funded work. This research was conducted as part of work performed under the CMS contract, and hence exempt from IRB approval as this contract falls under the classification of federal quality improvement and regulatory oversight.

We considered three survey outcome variables: presence of condition-level citations (indicating serious violations), total number of citations, and citation rate. Regarding citation numbers, we considered both types of citations—the serious condition-level citation (there are 16 in total) and the less serious standard citation (there are 367 in total). The total citation was defined as the sum of condition-level and standard-level citations. The citation rate was defined as the total number of citations identified per survey-hour, where the total survey-hours was the time spent by all team members at the dialysis facility during the survey.

Facility-level covariates included whether a facility was urban (as defined by the rural-urban commuting area (RUCA) code associated with the facility’s ZIP code), reuse of dialysate, number of patients, and modalities provided (hemodialysis versus peritoneal dialysis). Facility-level clinical outcomes included mortality (Standardized Mortality Ratio), hospitalization (Standardized Hospitalization Ratio of admissions) and hemodialysis access infection rate from the FY 2014 Dialysis Facility Reports. Finally, we used four survey-level data elements: total number of surveyors; total averaged on-site surveyor time spent on all previous surveys since 2009 (as a surrogate measure to represent surveyor’s experience); number of years since the facility was last surveyed; and number of surveys the facility has undergone since the new CfC ESRD regulations.

### Statistical analysis

We conducted propensity score analysis to reduce potential selection bias in assignment to the “experiment” [8]. We first estimate the propensity score, i.e., the probability of Core survey assignment, based on the pre-survey facility-level covariates and the facility’s state of residence using logistic regression models. Facilities with Core Surveys were then matched to those with Basic Surveys based on the estimated propensity scores through a “5-to-1 digit” greedy matching algorithm [9]. For each Core Survey, we identified a matching Basic Survey within the same state to reduce bias attributed to variation of state-level survey and oversight activities [10]. Unmatched surveys were excluded from the following analysis, a common practice in propensity matching analysis [7]. We assessed balance in the individual covariates after matching by comparing the means (or prevalences) of each facility- and survey-level covariate between the Core and Basic Survey groups and by examining their standard differences, i.e. means divided by standard deviations. A standard difference of < 0.1, which implies that the difference of the means between groups is 10% of the standard deviation, is commonly accepted as a negligible difference [11]. We also assessed the overall balance through plotting the survey-type-specific distributions of propensity scores and the Kolmogorov-Smirnov test, which tests for equal distributions. Similarly distributed propensity scores indicated balance in the distribution of facility-level covariates across the Core and Basic Survey groups [7].

We estimated the Core Survey effect using different types of regression models, depending on the type of outcome variable. First, the majority of facilities (>80%) had zero condition-level citations. We thus assessed a probability of having at least one condition-level citation and used a logistic regression model to estimate the Core Survey effect on identification of these citations. Second, for the counts and rate of total citations (condition and standard), we used a zero-inflated Poisson model (ZIP) [12–14] since there exist excessive zero counts (or, *structural zeros*) beyond what could be explained by random sampling from a Poisson distribution. Third, in all the above regression model estimations, we included a matched-pair-specific random intercept as well as facility and survey characteristics. The former was to account for the correlation within the matched pairs, and the latter was to adjust for residual confounding in the observed covariates [15].

## Results

There were 2,571 facilities surveyed between January 2013 and July 2014. We excluded one facility in New York due to incomplete citation counts and 185 facilities due to missing important facility-level covariates that were used in the matching method. The final sample was N = 2,385 facilities, of which 1,275 (53%) underwent Basic Surveys and 1,110 (47%) were surveyed with the Core Survey. In Table 1, we observed systematic differences in facility and survey characteristics between the two survey processes in the original unmatched sample. In particular, the Core Survey was more likely used in rural areas, when the survey team size was as small as one, and for facilities where 3.5 years or more had elapsed since their last survey. These factors could potentially lead to confounding in these results. Therefore, we took these into account in our modeling by utilizing the propensity score matching method, a statistical method specifically designed to reduce confounding biases in our setting.

**Table 1:** Comparison of baseline characteristics between Basic and Core surveys in the unmatched and matched samples. Std. Diff, Standard difference. Continuous variables are reported as mean ± standard deviation. Categorical variables are reported as number (%).

	Unmatched Sample		Matched Sample		
	Basic	Core	Basic	Core	
Baseline Variable	(N = 1275)	(N = 1110)	(N = 633)	(N = 633)	Std. Diff
Facility in Urban Area	976 (76.5%)	814 (73.3%)	491 (77.6%)	494 (78.0%)	0.009
Facility Practices Reuse	303 (23.8%)	289 (26.0%)	162 (25.6%)	159 (25.1%)	0.009
In-center Hemodialysis	1265 (99.2%)	1101 (99.2%)	629 (99.4%)	627 (99.1%)	0.028
Home Hemodialysis	348 (27.3%)	307 (27.7%)	159 (25.1%)	160 (25.3%)	0.003
Peritoneal Dialysis	635 (49.8%)	561 (50.5%)	308 (48.7%)	311 (49.1%)	0.008
Facility Size					0.024
<50 patients	388 (30.4%)	348 (31.4%)	201 (31.8%)	194 (30.6%)	
50-<100 patients	510 (40%)	464 (41.8%)	249 (39.3%)	252 (39.8%)	
100+ patients	377 (29.6%)	298 (26.8%)	183 (28.9%)	187 (29.5%)	
SHR in 2009–2012	1.015±0.269	1.023±0.264	1.026±0.271	1.035±0.259	0.034
SMR in 2009–2012	1.043±0.277	1.047±0.270	1.066±0.295	1.051±0.272	0.054
Infection Rate in 2012	2.000±2.232	2.024±1.656	2.003±1.604	2.109±1.834	0.061
Percent Tier 2 Facility in 2012	275 (21.6%)	266 (24.0%)	135 (21.3%)	144 (22.7%)	0.028
Percent Tier 2 Facility in 2013	392 (30.7%)	310 (27.9%)	182 (28.8%)	183 (28.9%)	0.003
Number of Surveyors					0.040
1	509 (39.9%)	501 (45.1%)	257 (40.6%)	245 (38.7%)	
2	511 (40.1%)	424 (38.2%)	266 (42.0%)	277 (43.8%)	
3+	255 (20.0%)	185 (16.7%)	110 (17.4%)	111 (17.5%)	
Total Surveyor's Historical Hours	29.00±16.18	28.57±16.69	29.55±16.26	29.92±16.65	0.022
Time Since Last Survey > 3.5 Years	448 (35.1%)	492 (44.3%)	234 (37.0%)	238 (37.6%)	0.011
Number of past surveys conducted since new ESRD regulations	1.958±0.529	1.932±0.570	1.964±0.543	1.956±0.538	0.015

SMR: Standardized Mortality Ratio; SHR: Standardized Hospitalization Ratio.

### Balance in facility characteristics between Core and Basic surveys

**Fig 1:**
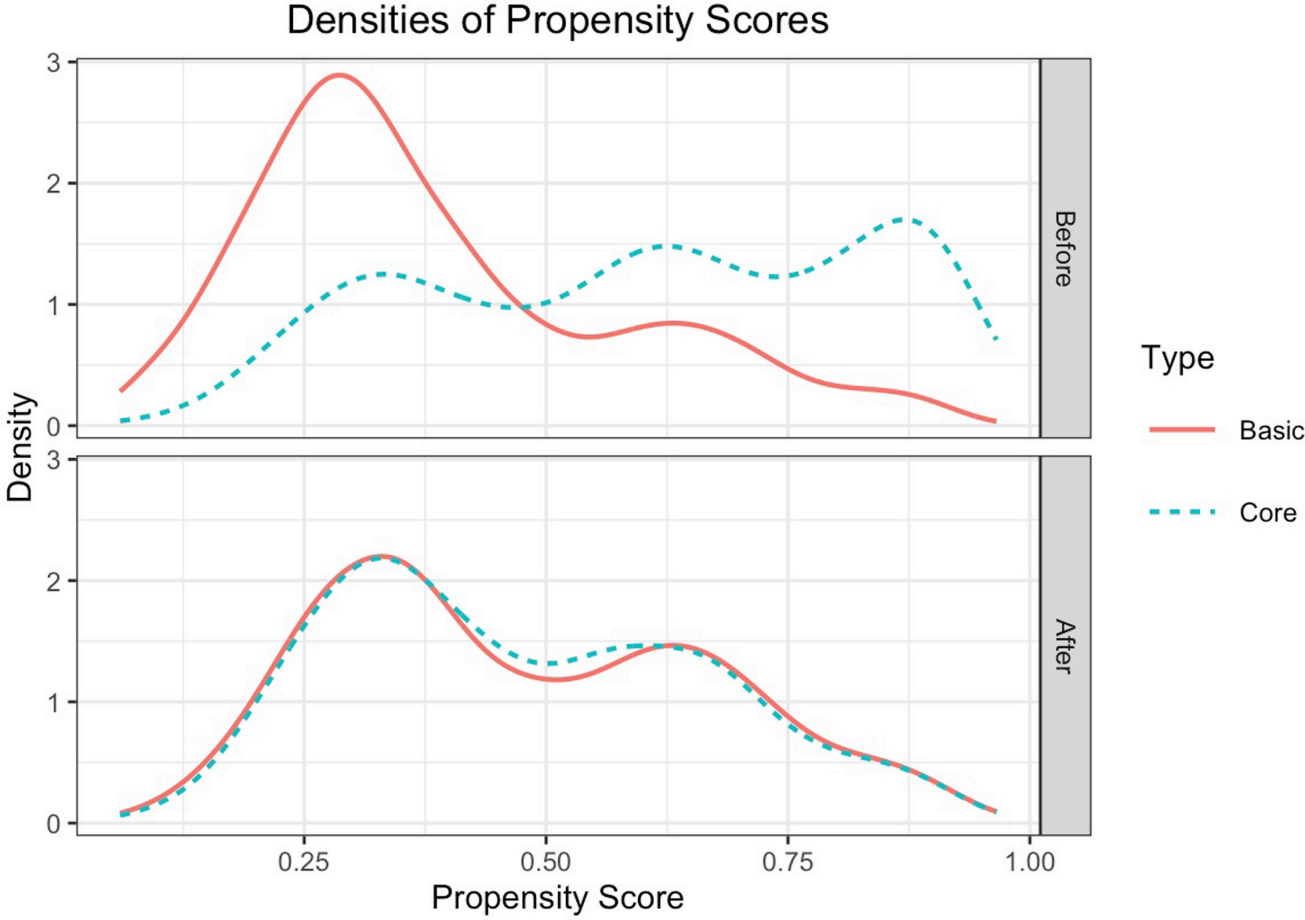
Distribution of the estimated propensity score by survey type in the original unmatched sample (top panel) and in the matched sample (bottom panel). Solid line corresponds to Basic survey. Dashed line corresponds to Core survey.

The propensity score matching algorithm resulted in a total of 633 within-state matched pairs (1,266 facilities). Table 1 reports the means (or percentages) of the individual baseline covariates for the Core and Basic Survey groups. After matching, we observed that many of the systematic differences in the original unmatched sample were diminished. Nevertheless, time since the facility’s last survey, the number of surveyors and the sum of surveyors’ total historical surveying time are important survey characteristics that likely play a critical role influencing survey outcomes. Hence, we used regression adjustment including all survey and facility characteristics to further reduce bias due to residual confounding [15]. The overall covariate balance between the two survey groups is shown in Fig 1. The top panel of Fig 1 shows the propensity scores were off-balance between the Basic and Core Surveys in the unmatched sample (P<0.001 based on the Kolmogorov-Smirnov test for equal distributions). However, balance was improved through the propensity score matching (Fig 1, bottom panel; P = 0.910 based on the Kolmogorov-Smirnov test).

### Assessing efficiency of the Core Survey

Examination of the unmatched results, not accounting for any facility and survey characteristics, suggested that the Core Survey appeared to identify more condition-level and standard-level citations than the Basic Survey, and resulted in a higher citation rate, although the difference in condition-level citations did not reach statistical significance (Table 2).

**Table 2:** Comparison of survey outcomes between Basic and Core surveys in the original unmatched sample. All outcomes are reported as mean ± standard deviation.

Outcome Variable	Basic (N = 1275)	Core (N = 1110)	P^*^
Citation Counts:			
Condition-level	0.32 ± 0.89	0.37 ± 1.02	0.200
Standard-level	3.97 ± 4.09	4.32 ± 4.07	0.001
Total	4.28 ± 4.63	4.69 ± 4.75	0.001
Citation Rate	0.13 ± 0.29	0.22 ± 1.58	<.001

* Wilcoxon’s rank-sum test was used.

Table 3 presents the adjusted odds/relative ratios of Core Survey use, compared with the Basic Survey, in the matched sample. In the matched sample, we found that the Core Survey had higher odds of identifying condition-level citations (26%), although the odds ratio was not statistically significant (P = 0.143). In the ZIP models for total citation counts and citation rate, the presence of structural zeros (excessive zero counts) reflected a significant portion of facilities that would not have any violations and hence were ‘not at risk’ for identification of citations. Because the proportion of structural zeros in facilities with the Basic Survey was higher, we needed to statistically account for these different proportions to fairly assess the relative effect of the Core Survey on the number (and rate) of citations. We estimated that 11% of facilities surveyed with the Core Survey were not at risk, while 19% of facilities surveyed with the Basic Survey were not at risk. Thus, if the structural zeros were not taken into account in our analyses, we would likely have observed an undercounting of total citations in facilities with the Basic Survey. This was shown in our preliminary analysis result with the unmatched sample where the total citation rate of facilities undergoing the Core Survey was about twice as high as those with the Basic Survey (Table 2). After accounting for the imbalanced proportions of the structural zeros (zero citations), we found that the Core Survey identified approximately 10% more total citations (P = 0.001). This translated to an increase of 10% in survey efficiency (defined as the number of total citations detected per survey-hour) compared to the Basic Survey (P = 0.002).

**Table 3:** Estimated odds ratio/relative ratio of Core Survey use, compared with the Basic Survey, in the matched sample. The estimates are adjusted for baseline facility and survey characteristics.

Outcome Variable	Odds/Relative Ratio	95% CI		P
Presence of Condition-level Citations (logistic model)	1.255	0.927	1.700	0.143
Total Citation Counts (ZIP–Poisson model)	1.102	1.040	1.167	0.001
Citation Rate (ZIP–Poisson model)	1.097	1.036	1.162	0.002

## Discussion

Federal regulatory oversight provides the policy context for our study results. Congressional investigation of dialysis facility oversight led to multiple calls to increase the frequency, consistency and breadth of the dialysis survey process. Our results suggest the Core Survey in the initial implementation period yielded greater efficiency, as measured by the rate of identifying citations (regulatory violations) per on-site hour compared to the Basic Survey. In our propensity score matched analyses, the Core Survey process identified 10% more total citations and was 10% more efficient than the Basic Survey. While the Core Survey resulted in identifying more condition-level citations, the absence of statistical significance for this result could be attributed to the overall observed lower event counts for these severe violations.

State Survey Agencies did not fully adopt the Core Survey at the same time, given the finite training resources available and duration of time needed to train several hundred State Surveyors, resulting in non-random assignment of the Core Survey to facilities. Because of this, the effect of the Core Survey on the outcomes was confounded by facility characteristics, health outcomes, past and current survey characteristics, and state ESRD regulations (present in approximately 50% of states). A major strength of this study is the use of propensity score matching where a matched pair was identified within states. Our approach attempted to adjust for observed variations between the two survey groups as well as to control for unobserved variation within states, such as states’ allocation of resources to the survey agencies, state-level policy climate for regulatory activities and current CMS regional office monitoring of state survey agencies. These important factors require further investigation in future research to more comprehensively assess the efficiency of the Core Survey nationally and to better characterize state- or regional-level variation. Another strength of our analytical approach is the use of zero-inflated Poisson (ZIP) models. The ZIP model enabled us to take into account the imbalance of ‘good’ (or ‘not-at-risk’) facilities for which zero violations would be observed regardless of the time spent for the survey. It is a crucial feature that cannot be ignored, especially when our research interest is in evaluating a citation rate per survey-hour. We acknowledge that an efficiency gain in the more focused and prescriptive Core Survey could be achieved as a result of spending less time at ‘good’ facilities and more time at ‘at-risk’ facilities. Accounting for a proportion of ‘good’ facilities as a latent factor, we were able to more directly quantify efficiency gains (or losses).

There are also a few limitations in our analysis. First, there were likely unmeasured confounders present, despite our attempts at mitigating confounding via careful study design. Second, identification of the survey process used for each included survey used an interim reporting approach; in a national memo, SCG instructed surveyors to write “CORE” in the summary findings field of the CMS Form 2567, which was how we distinguished Core from Basic surveys during the transition period in 2013 and 2014. During the data collection period, we found that a few surveys were mislabeled, but they were corrected prior to the analysis. It is possible some surveys assumed to be Basic may have been Core but were not identified as such.

Our study is a first step in examining the impact of changes in the dialysis facility oversight process under the ESRD dialysis facility. Because there is a dearth of published research on dialysis facility regulatory oversight, we draw on studies examining nursing home oversight. Both healthcare settings have several characteristics in common. Oversight activities face similar state-level pressures for resources. Nursing homes are also subject to reporting of quality outcomes and have been the subject of Congressional concerns about insufficient state survey agency monitoring and poor care quality of residents. This attention resulted in calls for and subsequent substantial changes in oversight, including the authority to impose monetary penalties against nursing homes. Moreover, both healthcare settings primarily serve Medicare beneficiaries; several private for-profit corporations own a majority of the facilities; state survey agencies are responsible for oversight since Medicare requires certification for reimbursement purposes; and the foci of surveys include clinical quality outcomes. These similarities provide a reasonably comparable framework for assessing our results.

In the nursing home regulatory setting, multiple studies have found that facilities providing poor quality of care in a particular domain were more likely to receive deficiency citations. For example, facilities with lower staffing levels were more likely to receive more deficiencies [16–18]. Additionally, more stringent oversight and greater state health department resources allocated to nursing home oversight were associated with improved quality outcomes and identification of more deficiencies [19]. While state-level resources affect nursing home survey activities, so too does greater federal regulatory guidance, which was found to improve the consistency and performance of licensing and oversight activities [20]. Other factors influencing nursing home oversight include whether governors were Democrats; percentage of chain facilities in the state; nursing home occupancy rates; and various other social, political, and economic factors [21–22]. For example, states with Democratic governors were more strict in enforcement of standards, and states with larger portions of for-profit facilities and states with lower nursing home occupancy rates, respectively, tended to give a higher number of deficiencies to nursing homes [21]. While we did not examine these factors, it is possible they could have similar effects on the ESRD dialysis facility survey process.

Our findings suggest the higher total citation count and the higher citation rate can be attributed to the new ESRD Core Survey that relies on formal pre-survey procedures and worksheets, as well as more streamlined on-site assessment. The more comprehensive and prescriptive structure of the Core Survey better focuses surveyor time on targeting critical data-driven areas such as mortality, infection control and prevention, medical error and adverse events, and QAPI activities, rather than spreading their time across a broader set of outcomes where the facility may be meeting most regulatory standards. The Core Survey increases survey effectiveness without added resource use. Furthermore, the timing of our evaluation compares the two processes shortly after implementation of the new Core Survey. It is reasonable to assume that there is a “learning curve” that might result in underestimation of the time savings inherent in this new survey process. Follow-up studies are needed. If they demonstrate that the Core Survey approach results in more cost effectiveness, then broader implementation of the Core Survey principles into survey processes for other care settings may be warranted.

## Supporting information

S1 Table Comparison between the Basic and Core process. (DOCX)

## Abbreviations

ESRD: end-stage renal disease
CMS: Centers for Medicare and Medicaid Services
CfC: Conditions for Coverage
FY: fiscal year
SCG: Survey and Certification Group
QAPI: Quality Assurance and Performance Improvement
