# Supplementary material for: Comparative effectiveness analysis of Medicare dialysis facility survey processes
Source: PLoS One. 2019 Apr 26;14(4):e0216038. doi: 10.1371/journal.pone.0216038 (PMC6485704; doi:10.1371/journal.pone.0216038)
Supplement: S1 Table — (DOCX) [file pone.0216038.s001.docx]

**S1 Table: Comparison between the Basic and Core process**

This table compares the tasks between the Basic and Core process. In general, the Core process includes a smaller, more refined set of questions/required observations than the Basic process. If “no changes” are noted in the Core process column, then the Core survey does not deviate in content and structure from the Basic survey, although it will include fewer questions/required observations. More detailed Outline of ESRD survey process can be found from the Centers for Medicare and Medicaid Services website: https://www.cms.gov/Medicare/Provider-Enrollment-and-certification/guidanceforlawsandregulations/dialysis.html.

| **Survey task** | **Basic survey** | **Changes in the Core survey** |
| --- | --- | --- |
| 1. Presurvey preparation | - Decide how to focus the survey using (a) specific aspects of the facility and (b) patient outcome measures from the facility’s latest Dialysis Facility Report. - Develop specific questions for the patient and staff interviews | No changes |
| 2. Introductions at the facility | At the facility, introduce and explain purpose of the visit | No changes |
| 3. Tour and observations of care   1. Environmental tour 2. Observation of HD patient care 3. Emergency equipment and preparation | Conducted almost immediately upon entering the facility   1. Generally inspect all areas of care – waiting rooms, patient restrooms, treatment areas (chairs, drip chambers), waste storage, etc. 2. Observe dialysis treatments 3. Ensure emergency equipment is functional | Collapsed into “Environmental ‘Flash’ Tour,” which involved observing four areas and asking staff in each area about culture of safety:   - In-center HD treatment - Water treatment/ dialysate preparation - Reuse room - Home dialysis training |
| 4. Entrance conference | Discuss focus areas developed during presurvey preparation with facility personnel (e.g., review how their facility infection rate compares with the state and US rates). Request internal facility documentation of quality improvement and any information needed for patient interviews and medical record review (see Task 5) | No changes |
| [NEW for Core] Observations of hemodialysis care & infection control practices | N/A | Observe staff providing care to HD patients and document anomalies via a set of infection control checklists |
| 5. Patient sample selection | Select 10% of patient population, attempting to choose all modalities and focusing on patients who appear to be outliers (e.g., repeated hospitalizations, low hemoglobin). | Same as the Basic process, but specifically include:   - Unstable patients - New admissions - Long-term care residents - Patients observed during Task 3 with concerns (e.g., alarms not answered, experienced dizziness) - Patients involuntarily discharged in the past year (records review only) - If applicable, patients involved in a complaint |
| 6. Water treatment and dialysate preparation | Observe water treatment equipment, water treatment logs, and dialysate preparation/delivery. Review protocol with relevant staff. | No substantial changes |
| 7. Dialysate reuse | Observe reuse/reprocessing room and equipment, review reuse logs, and, if applicable, observe centralized reprocessing. Review protocol with relevant staff. | No substantial changes |
| 8. Dialysis equipment maintenance | Interview personnel, review machine maintenance and repair logs, review 12-month culture results, | No changes |
| 9. Home dialysis | Observe the home training area and interview home training nurse(s) | No changes |
| 10. Patient interviews | Interview as many patients as possible among those identified in Task 5 | Must interview at least 4 patients |
| 11. Medical record review | Review all records for patients selected in Task 5. | Additional focus on prescription and medication orders, as well as the response by the patient’s Interdisciplinary Team in instances of outlier outcomes |
| 12. Personnel interviews | Interview personnel across all functions – medical director, social worker, reuse technicians, etc. | No changes |
| 13. Quality assessment and performance improvement | Review facility’s internal documents provided in Task 4 and, if available, look at facility’s four-year trends in outcomes on latest Dialysis Facility Report | Focus on areas identified during presurvey preparation; mandatory review of rates/trends in the facility’s mortality and incidence of infection; deeper assessment of the facility’s culture of safety, including risk identification and patient engagement |
| 14. Personnel record review | Review files of a cross-section of staff members—nurses, dietitians, water technicians, etc. | Minimum of three personnel |

**References**

Department of Health and Human Service. Centers for Medicare & Medicaid Services. Center for Clinical Standards and Quality/Survey & Certification Group. Revised Roll-Out of the New End Stage Renal Disease (ESRD) Core Survey Process. S&C memo 13-18-ES. March 8, 2013. <https://www.cms.gov/Medicare/Provider-Enrollment-and-Certification/SurveyCertificationGenInfo/Downloads/Survey-and-Cert-Letter-13-18.pdf>. Accessed December 23, 2018.
